# Chronique d’une mort annoncée : le destin funeste des disciplines de médecine tropicale françaises et ses conséquences dans la lutte contre les inégalités sanitaires en Afrique

**DOI:** 10.48327/mtsi.v2i1.2022.221

**Published:** 2022-02-25

**Authors:** Pascal MILLET

**Affiliations:** PhD, consultant senior, ancien chercheur hospitalo-universitaire en parasitologie, CHU de Bordeaux, France

**Keywords:** Médecine tropicale, Inégalités sanitaires, Histoire de la médecine, France, Afrique, Tropical medicine, Health inequalities, History of medicine, France, Africa

## Abstract

Depuis la fin des années 1990, nous assistons en France à une évolution lente conduisant à la disparition des disciplines liées aux maladies tropicales. Au niveau hospitalier, elle se traduit par la dilution progressive des services cliniques consacrés au traitement des maladies infectieuses et tropicales au sein d’un pôle infectieux ou de médecine interne, et, en biologie médicale, par le remplacement des biologistes parasitologues ayant acquis une spécialisation en mycologie, par des biologistes mycologues ayant acquis une spécialisation en parasitologie. Cette orientation peut sembler normale, la réduction des compétences en diagnostic parasitologique et clinique étant liée au succès des mesures d’hygiène et de contrôle des produits alimentaires, qui ont entraîné la quasi-disparition, dans notre pays, de parasitoses autochtones telles que la fasciolose, le tœniasis, ou l’amibiase. Priorité est donc donnée à la mycologie, en particulier aux infections respiratoires, prédominantes dans une population protégée et vieillissante. Fallait-il pour autant le faire au détriment des compétences en diagnostic et en soin des maladies touchant les populations des pays à ressources limitées ?

## Abstract (en)

Since the end of the 1990s, we have witnessed a slow evolution in France leading to the disappearance of disciplines related to tropical diseases. At the hospital level, this is reflected by the progressive inclusion of clinical services dedicated to the treatment of infectious and tropical diseases within an infectious diseases or internal medicine pole, and, in medical biology, by the replacement of parasitological biologists having acquired a specialization in mycology, by mycological biologists having acquired a specialization in parasitology. This orientation may seem normal, the reduction of skills in parasitological and clinical diagnosis being linked to the success of hygiene and food control measures, which have led to the virtual disappearance, in our country, of autochthonous parasitic diseases such as fasciolosis, taeniasis, or amoebiasis. Priority is therefore given to mycology, especially respiratory infections, which are predominant in protected and aging populations. However, should this be done at the expense of diagnostic and treatment skills for diseases affecting populations in countries with limited resources?

## Disparition des compétences par abandon de la formation en France

Marginaliser, dans les facultés de médecine, les compétences en parasitologie clinique et diagnostique, en réduisant drastiquement les heures d’enseignement consacrées aux maladies tropicales, et en faisant disparaître les travaux dirigés et pratiques, a détourné l’intérêt des étudiants pour ces disciplines. La raréfaction des postes cliniques et biologiques dans ces disciplines y a aussi largement contribué.

Cette disparition des postes va de pair avec la fermeture des instituts de médecine tropicale des villes françaises, comme l’Institut de médecine tropicale du Service de santé des armées (appelé École du Pharo) en 2013 à Marseille, qui formait tout autant des médecins français civils et militaires à l’exercice de la médecine au-delà des mers que des médecins africains qui retournaient ensuite pratiquer dans leur pays, certains y occupant des postes à haute responsabilité. Les médecins français pouvaient acquérir au Pharo des compétences cliniques pour le traitement des maladies dans les pays à ressources limitées. Les liens créés pendant ces formations assuraient le maintien de collaborations médicales et de recherche entre la France et les pays francophones d’Afrique ou d’Asie, à partir d’un réseau entretenu par le ministère de la Coopération d’alors. De même, la fermeture en 2011 de l’École de Santé Navale à Bordeaux, ville où la médecine tropicale a joué un rôle crucial dans les actions de formation et de coopération, est un exemple frappant de la ligne de conduite française vers l’éradication de la discipline. Ainsi Bordeaux pensa-t-elle se débarrasser de son image de ville portant la tache indélébile des colonisations et du rôle qu’elle joua dans le triangle du marché des esclaves au XVII^e^ siècle. La suppression d’institutions ne fera pourtant pas oublier l’histoire, et la restructuration d’une coopération régionale française équilibrée avec certains pays africains aurait été plus efficace.

## Abandon par les pays indépendants des infrastructures sanitaires et de la formation

Il appartient aux historiens de décider de l’impact de la colonisation et, ensuite, de la coopération française, sur le développement de la santé publique des pays africains et asiatiques concernés. Si l’on regarde aujourd’hui le fonctionnement de la pyramide sanitaire des pays francophones africains, nous ne sommes pas convaincus d’un rôle positif de la coopération française sur le long terme. Pourtant, j’ai eu l’occasion d’observer, dans les années 1990 au Gabon, le fonctionnement des derniers centres de santé dirigés par des médecins militaires français dans les villes de Mouila et de Franceville. Les populations s’y rendaient et étaient soignées gratuitement. Les hôpitaux publics gabonais, en revanche, bénéficiaient déjà de programmes de coopération fortement dégradés, et se retrouvaient dans une situation déplorable.

Concernant la formation, il faut avouer que, contrairement à l’implication des Britanniques dans leurs ex-colonies, la France n’a pas poursuivi son investissement pédagogique dans les siennes. Il en résulte une différence notoire dans la qualité actuelle de l’enseignement dans les universités entre l’Afrique anglophone de l’Est et l’Afrique francophone Centrale et de l’Ouest. Le tissu relationnel hospitalo-universitaire franco-africain était pourtant très fort jusque dans les années 1980. Mais son délitement a été extrêmement rapide, notamment du fait d’un manque d’implication de la France et des États concernés dans l’administration des établissements universitaires et hospitaliers. Le Mali l’a bien compris, et s’est tourné vers les États-Unis pour développer un tissu scientifique et médical de qualité malgré une grande instabilité politique (www.niaid.nih.gov/research/mali-university-clinical-research-center).

## Une réorientation des priorités de santÉ vers l’intérêt mondial au détriment des populations locales

La France a progressivement réduit son intervention dans le fonctionnement des pyramides sanitaires des pays. La médecine tropicale du siècle dernier n’aurait-elle pas pu évoluer, au sortir de la coopération technique, vers un soutien à la prise en charge de la santé des populations à ressources limitées pour les maladies transmissibles et non transmissibles ? Les instituts de santé publique auraient pu le faire, mais les financements publics internationaux se sont rapidement concentrés verticalement sur les 3 maladies identifiées comme mondialement prioritaires dans les années 1990: le sida, le paludisme et la tuberculose [2]. Tellement concentrés que, dans plusieurs pays délaissant les questions de santé publique, il faut encore être atteint de l’une de ces trois maladies pour avoir l’espoir d’être pris en charge par des structures artificiellement créées hors d’une pyramide sanitaire déficiente, et de survivre. L’apparition du COVID-19 a conduit les bailleurs internationaux à revoir leurs priorités, mais nous restons dans la verticalisation des programmes de santé (en dépit d’une communication affichée à vouloir renforcer les systèmes de santé), et loin de la mise en place d’une prise en charge de la santé globale des populations des pays qui n’ont pas les moyens économiques de l’assurer.

En France, le recentrage de la médecine tropicale hospitalière vers les centres de santé pour les voyageurs se rendant dans les pays tropicaux, a détourné les collaborations privilégiant au départ la santé publique dans les pays africains, vers la santé du voyageur occidental en Afrique. De ce fait, les coopérations établies de longue date entre la France et les pays francophones africains se sont raréfiées, et ont été remplacées par des programmes de médecine préventive. Indirectement, la recherche sur les maladies tropicales, qui reposait sur des collaborations entre médecins tropicalistes français et africains, s’est retrouvée marginalisée, surtout suite à la disparition, en France, des instituts de médecine tropicale tels que ceux qui persistent toujours en Belgique (Institute of Tropical Medicine d’Anvers), en Suisse (Swiss TPH à Bâle), au Royaume-Uni (London School of Hygiene & Tropical Medicine), et dans de nombreux autres pays européens, avec des réorientations stratégiques en lien avec le monde d’aujourd’hui. Seul subsiste en France l’Institut Pasteur et son réseau d’instituts à l’étranger, qui privilégie toujours une recherche fondamentale malgré une santé publique déficiente, avec quand même un investissement notoire en biologie médicale, souvent en lien avec la Fondation Mérieux.

## Le devoir de non-ingérence au détriment de la vie

Le phénomène migratoire entre les continents africain et européen que nous vivons actuellement est, en partie, le résultat d’un abandon de l’accompagnement au développement des pays africains au nom de la non-ingérence. Eu égard à ce principe, des personnes meurent dans l’indifférence internationale. Au nom du même principe, nous laissons des ONG intervenir avec leurs propres moyens pour coller des pansements sur un terrain politique instable et déficient. Pourtant, les compétences techniques locales sont à présent en place. Des techniciens, médecins, biologistes très bien formés cherchent à améliorer la situation dans leurs pays, souvent dans des conditions impossibles de famine, de guerres, de manque d’infrastructures routières et d’éloignement géographique des capitales. Il manque à ces personnes un leadership gouvernemental, et le soutien d’une administration compétente capable de respecter la pyramide sanitaire et d’établir une organisation rationnelle de la prise en charge des soins. En 2020, l’OMS/Europe a délivré des conseils aux États membres sur le renforcement des systèmes de santé et la réorganisation rapide de la prestation de services afin de lutter contre le COVID-19 tout en maintenant les services essentiels de base dans le continuum de soins afin que personne ne soit laissé de côté [3]. Un rapport propose aux politiques une vision de la gouvernance pour la santé au XXI^e^ siècle [1]. Les écrits peuvent servir de support à une réflexion approfondie sur la manière d’appréhender une nouvelle forme de coopération.

## Vers une nouvelle diplomatie: moins de discours et de fierté, plus de concret

Au lieu de se contenter de twitter des propos superficiels - ce qui est devenu le moyen de communication le plus utilisé, y compris par des dirigeants politiques capables de bouleverser l’économie mondiale par quelques réflexions basiques - il est urgent de se pencher avec les pays africains sur les priorités de santé publique, et de réfléchir sérieusement à la mise en place d’une collaboration internationale équilibrée sur le sujet. Pour cela, nous devons considérer que l’histoire est derrière nous et que son déroulement ne peut être modifié. Au-delà du devoir de repentance, qui a déjà blessé profondément des hommes et des femmes ayant œuvré avec leur cœur et leur expertise pour améliorer la santé des populations, nous avons besoin d’un monde diplomatique fort, capable de s’entretenir avec les chefs d’État sur l’intérêt économique de prendre soin de leurs populations et d’assurer ainsi une croissance progressive de leur niveau de vie. Pourquoi la France, qui a porté les droits de l’homme dans l’histoire du monde, signé la première convention de Genève en 1864 instituant la Croix-Rouge et le droit international humanitaire à l’initiative du Suisse Henry Dunant, et instauré la sécurité sociale pour tous en 1945, ne pourrait-elle pas faire de la santé une priorité au lieu d’engager des médecins dans les ambassades comme spécialistes en communication autour des financements considérables que nous procurons au Fonds mondial de lutte contre le sida, la tuberculose et le paludisme, et dont seule une fraction minime revient à l’expertise française ?

L’histoire de notre pays en matière de santé et l’émulation qu’elle a pu donner à d’autres pays n’équilibrent-elles pas le devoir de remords prôné actuellement par nos politiciens ? Regrets et désintérêts ne doivent pas conduire une politique extérieure. Si nous voulons faire face à la fois à notre passé et à un présent pluriethnique, il est grand temps de tisser à nouveau des liens étroits avec les pays qui restent demandeurs d’une coopération saine et équilibrée. La santé pour tous est un des axes prioritaires que nous savions gérer, et un atout indispensable pour assurer le développement économique d’un pays.

## Liens d’intérêts

L’auteur ne déclare aucun lien d’intérêt.
